# Complex regional pain syndrome after distal radius fracture: A survey of current practices

**DOI:** 10.1371/journal.pone.0314307

**Published:** 2024-11-21

**Authors:** Alice (Wei Ting) Wang, Kelly A. Lefaivre, Jeffrey Potter, Aresh Sepehri, Pierre Guy, Henry Broekhuyse, Darren M. Roffey, David J. Stockton

**Affiliations:** 1 Faculty of Medicine, Department of Orthopaedics, University of British Columbia, Vancouver, British Columbia, Canada; 2 Division of Orthopaedic Trauma, Vancouver General Hospital, Vancouver Coastal Health, Vancouver, British Columbia, Canada; Southern Medical University Nanfang Hospital, CHINA

## Abstract

**Introduction:**

Complex regional pain syndrome (CRPS) is a common complication following distal radius fractures that is difficult to diagnose and can lead to permanent disability. While various proposed prophylaxis and treatment modalities exist, high-quality evidence guiding practice is limited. This survey of Orthopaedic Trauma Association (OTA) and Canadian Orthopaedic Association (COA) members was conducted with the primary aim of assessing practice patterns in distal radius fractures complicated with CRPS.

**Methods:**

An electronic survey was distributed to practicing orthopaedic surgeons in the COA and OTA. Questions assessed practice setting, preference in management of distal radius fractures and CRPS, comfort level in managing CRPS, and identification of gaps in management. Responses were anonymized and collected over 8 months. Response data was analyzed using descriptive statistics; thematic analysis was used on free text response.

**Results:**

134 survey responses were completed. 84% of respondents felt the incidence of CRPS in distal radius fractures was 1–10%, while 15% felt it was closer to 11–20%. 24% of respondents utilized the “Budapest Criteria” to diagnose CRPS. 40% offered prophylaxis in patients felt to be at high risk of developing CRPS. 66% of surgeons felt neutral, uncomfortable, or very uncomfortable managing CRPS in distal radius fractures. When asked to consider adopting a prophylactic therapy, 38% of surgeons indicated that a therapy that reduced the absolute risk of CRPS by 6–10% would change their practice. Gaps in current practice included lack of evidence-based treatment and prevention strategies and diagnostic uncertainty.

**Conclusion:**

This study identified that amongst orthopaedic surgeons in the COA and OTA, diagnosis, treatment, and prophylaxis strategies for CRPS in distal radius fractures are heterogeneous. Surgeons are not confident in their treatment of CRPS. Future studies using rigorous research methods are warranted to improve management.

## Introduction

Distal radius fractures are the most prevalent adult fracture, accounting for 17.5% of all fractures [1]. Complex regional pain syndrome (CRPS) is common in adult patients with distal radius fractures, with a reported incidence of up to 32% [2–5]. CRPS can lead to permanent disability, unemployment, and is costly to the patient and to the healthcare system, with an estimated cumulative outpatient and pain prescription cost of $42,026 over 8 years after diagnosis [6,7]. Even with the validation of the Budapest CRPS diagnostic criteria in 2010 [8], diagnosing CRPS can be a prolonged process due to the range of vague symptoms on presentation, which may lead to a delay in treatment and worsening of outcomes. Additionally, some of the features of the diagnostic criteria are part of the normal inflammatory response to fracture which makes it difficult for the practitioner to decide which diagnosis to attribute the symptom to–the fracture or superimposed CRPS.

The pathogenesis of CRPS is complex and incompletely understood; evidence suggests nervous system sensitisation, autonomic dysfunction, and inflammatory changes [9]. While there have been various proposed prophylaxis and treatment modalities, little high-quality evidence supports their efficacy. These include but are not limited to: oral anti-depressants, parenteral lidocaine and corticosteroids, surgical treatment with compressed nerve release, counselling, and occupational and physical therapy [10,11]. Vitamin C has been proposed as an effective prophylaxis for CRPS in distal radius fractures but data have been conflicting [12–16], with the most recent randomized controlled trial (RCT) in 2014 by Ekrol et al. showing no difference in functional outcomes or rate of CRPS in patients with distal radius fractures given oral Vitamin C versus placebo [14].

Given the lack of diagnostic and management guidelines in literature, we sought to survey orthopaedic surgeons on their experiences and preferences in managing patients with distal radius fractures and CRPS complications. Specifically, we aimed to assess the diagnostic criteria for CRPS used amongst surgeons as well as the management strategies in treatment and prophylaxis. The goals are to better understand current practices and identify knowledge gaps to guide future research focus.

## Methods

### Study design

The study protocol was conducted in accordance with the Strengthening the Reporting of Observational Studies in Epidemiology (STROBE) guidelines. Ethics approval was obtained from The University of British Columbia (UBC) Clinical Research Ethics Board (H23-00644). After identification of the research question, six experts in orthopaedic trauma were recruited to assist with the development of the questionnaire. Through an iterative process of feedback from this panel of surgeons, consensus was reached on the survey questions.

The survey was uploaded onto the UBC Qualtrics platform and distributed to members of the Canadian Orthopaedic Association (COA) and the Orthopaedic Trauma Association (OTA) by email. A link to the survey was also posted on the OTA website under the Research Surveys page. As outlined in the Letter of Initial Contact and then again in the study Consent Form, informed consent was obtained in the context of administering the survey. If the respondent clicked on the link to open the survey, proceeded to answer the questions, and pressed “SUBMIT” to upload their responses, then their informed consent was considered to have been provided. Participants who clicked on the link were told they could choose to pull out of the study prior to submission by closing their internet browser, but they were not able to withdraw after their survey responses had been submitted.

### Outcome measures

An electronic-based 23-question survey was created with questions on surgeon’s practice setting, preference for management of distal radius fractures, diagnostic criteria used to diagnose CRPS, perception of CRPS incidence in distal radius fractures, preference for prophylaxis and treatment options for CRPS in distal radius fractures, surgeon’s comfort level in managing CRPS, and gaps surgeons identify in management of this patient population. The question format included multiple-choice and free text response (S1 File). Survey responses were anonymized and collected over an 8-month period from 1 May 2023 to 6 January 2024.

### Data preparation

Only surveys with all questions completed were included for data analysis. Response data was analyzed using descriptive statistics. Thematic analysis was performed on free text responses. Codes were generated from all free text responses in a systematic manner to enable categorization and were subsequently grouped into themes. Identified themes were then reviewed, modified, and finalized.

## Results

**Fig 1:**
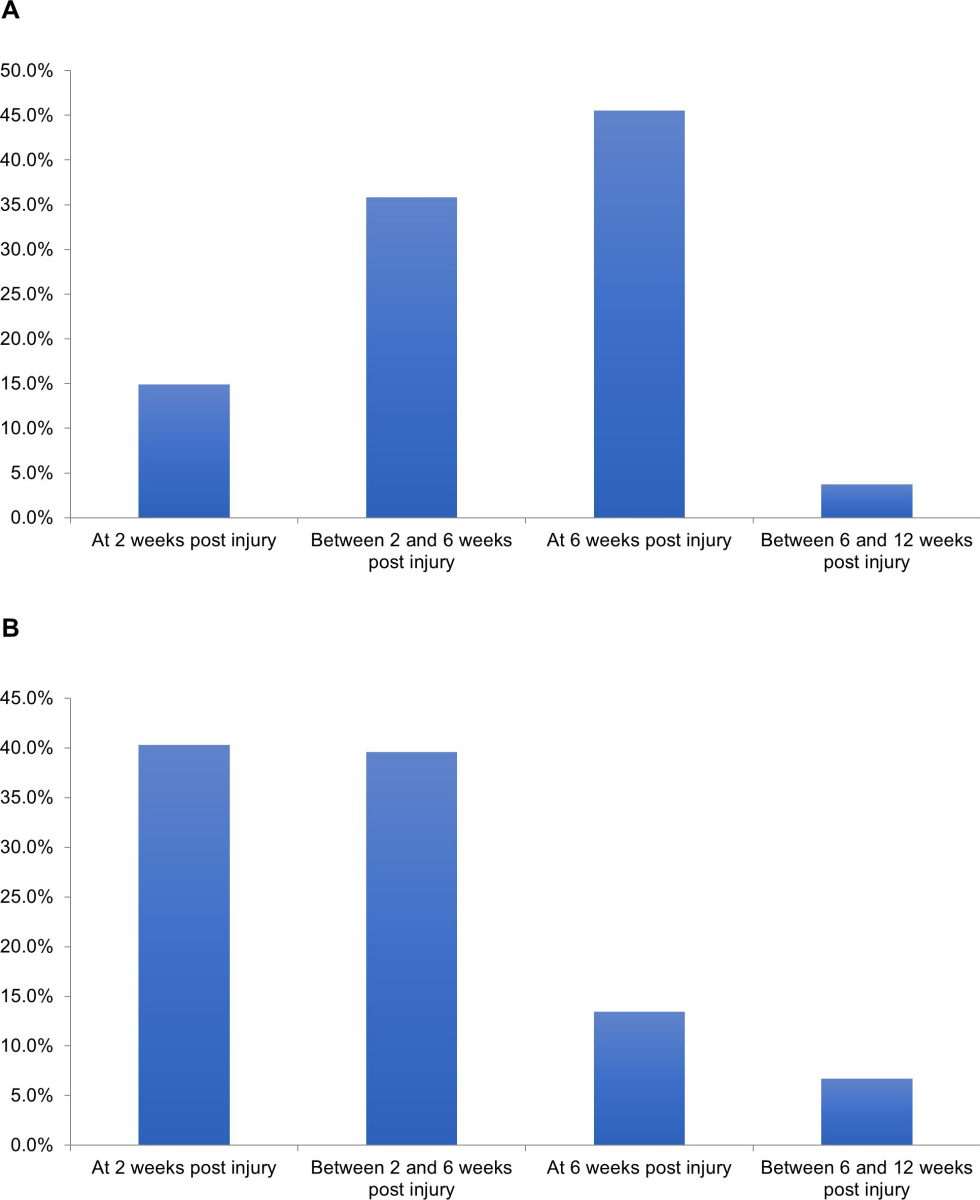
Time when partial weightbearing is allowed (e.g., perform activities of daily living) in A) non-operatively treated and B) operatively treated distal radius fractures.

One hundred and sixty-nine surveys were started, with 134 complete responses. Sixty-six percent of surgeons practiced in Level one/two or tertiary/quaternary centres, with 56% primarily practicing trauma and 27% with hand and wrist subspecialty training (S1 Fig). Eighty-eight percent of respondents treated patients in a circumferential cast for non-operative distal radius fractures, while 85% immobilized operatively treated distal radius fractures in a splint. Patients were allowed to partially and fully weight bear to the affected upper extremity at various time intervals in both non-operatively and operatively managed cases (Fig 1). Range of motion exercises to the affected wrist started at 6 weeks for 75% of respondents in non-operatively treated distal radius fractures. In contrast, for operatively treated distal radius fractures, 53% and 34% of respondents began range of motion exercises at 2 weeks or between 2 to 6 weeks post-operatively, respectively.

**Fig 2:**
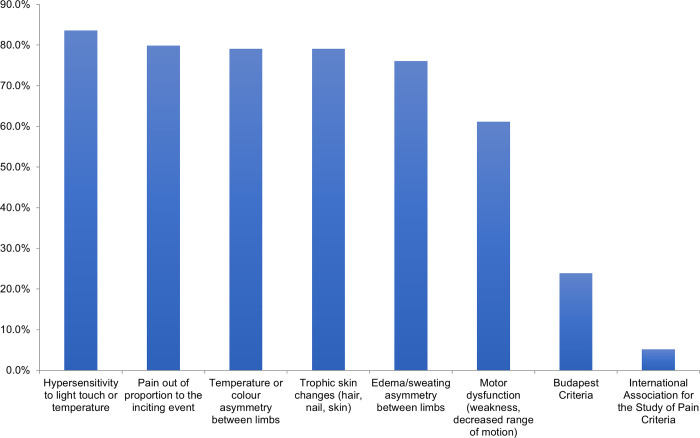
Features used to diagnose CRPS in distal radius fractures.

Eighty-four percent of respondents estimated the incidence of CRPS in distal radius fractures to be 1–10%, 15% of respondents felt it was closer to 11–20%, and 1% felt it was higher. The most common feature used to diagnose CRPS is “hypersensitivity to light touch or temperature”, followed by “pain out of proportion”, “temperature or colour asymmetry between limbs” and “trophic skin changes” (Fig 2). Only 24% of respondents used the Budapest Criteria for diagnosing CRPS [17].

**Fig 3:**
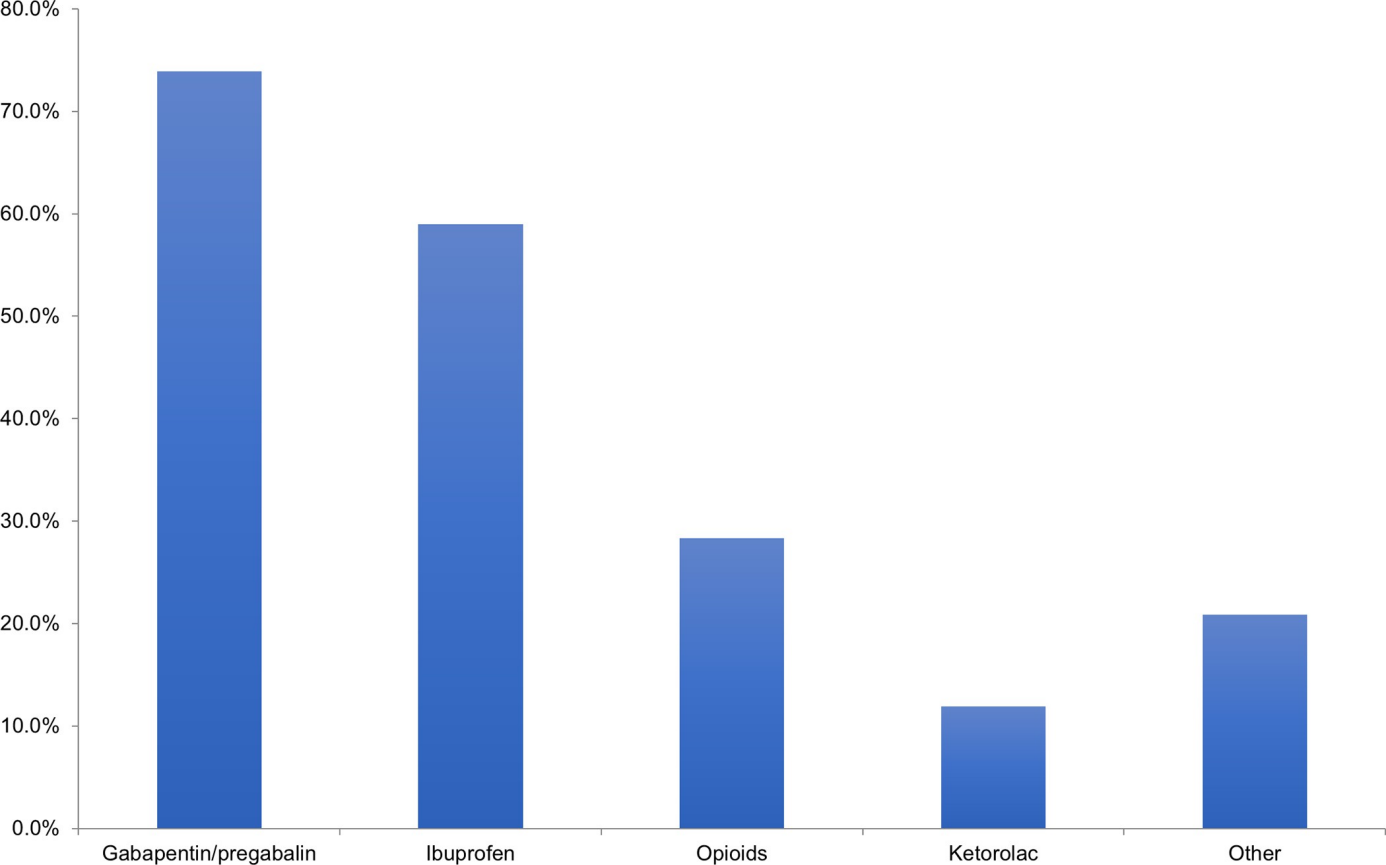
Analgesic medications prescribed in patients diagnosed with CRPS in distal radius fracture.

**Fig 4:**
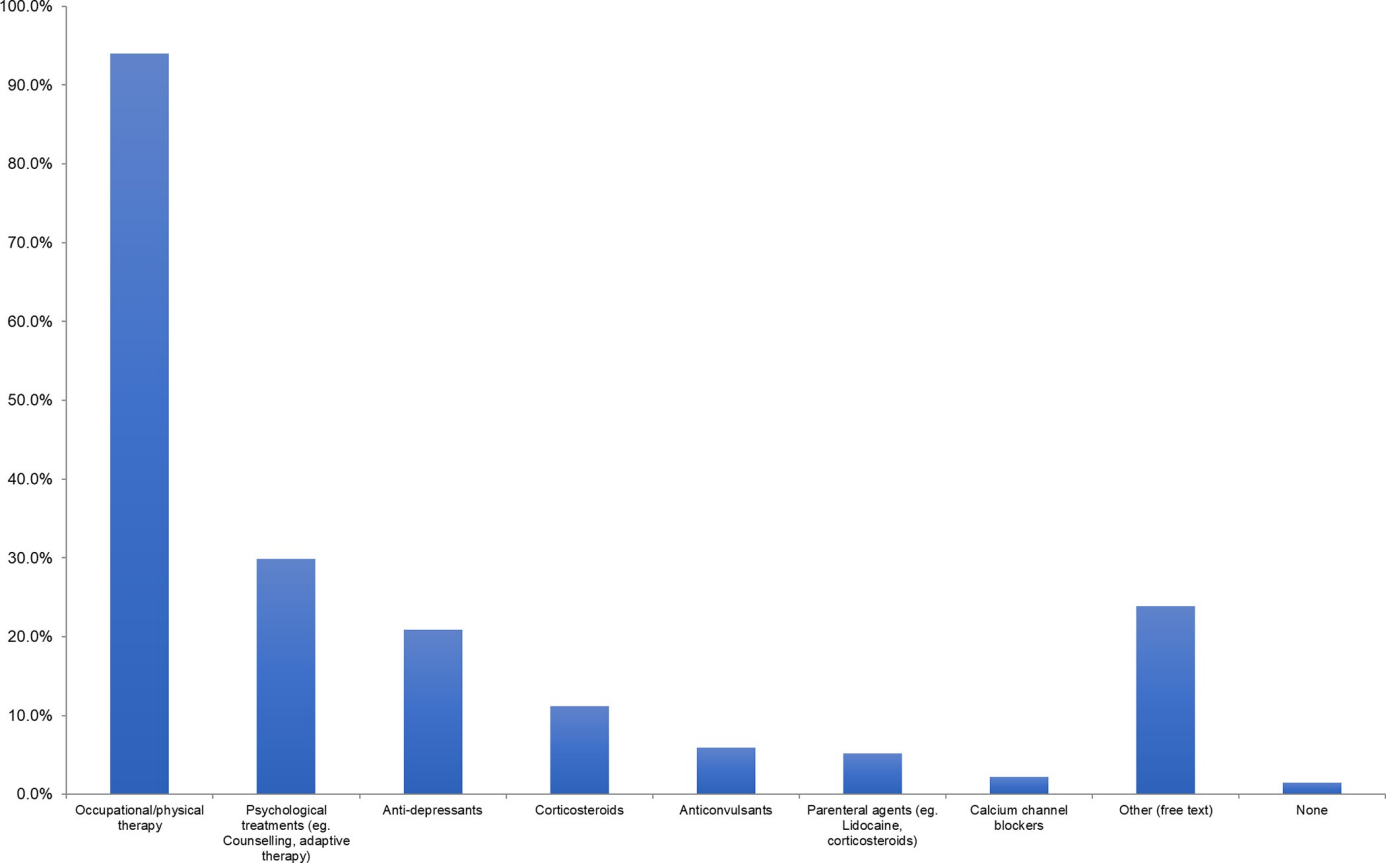
Additional treatment modalities offered to or referred for patients diagnosed with CRPS in distal radius fracture.

**Fig 5:**
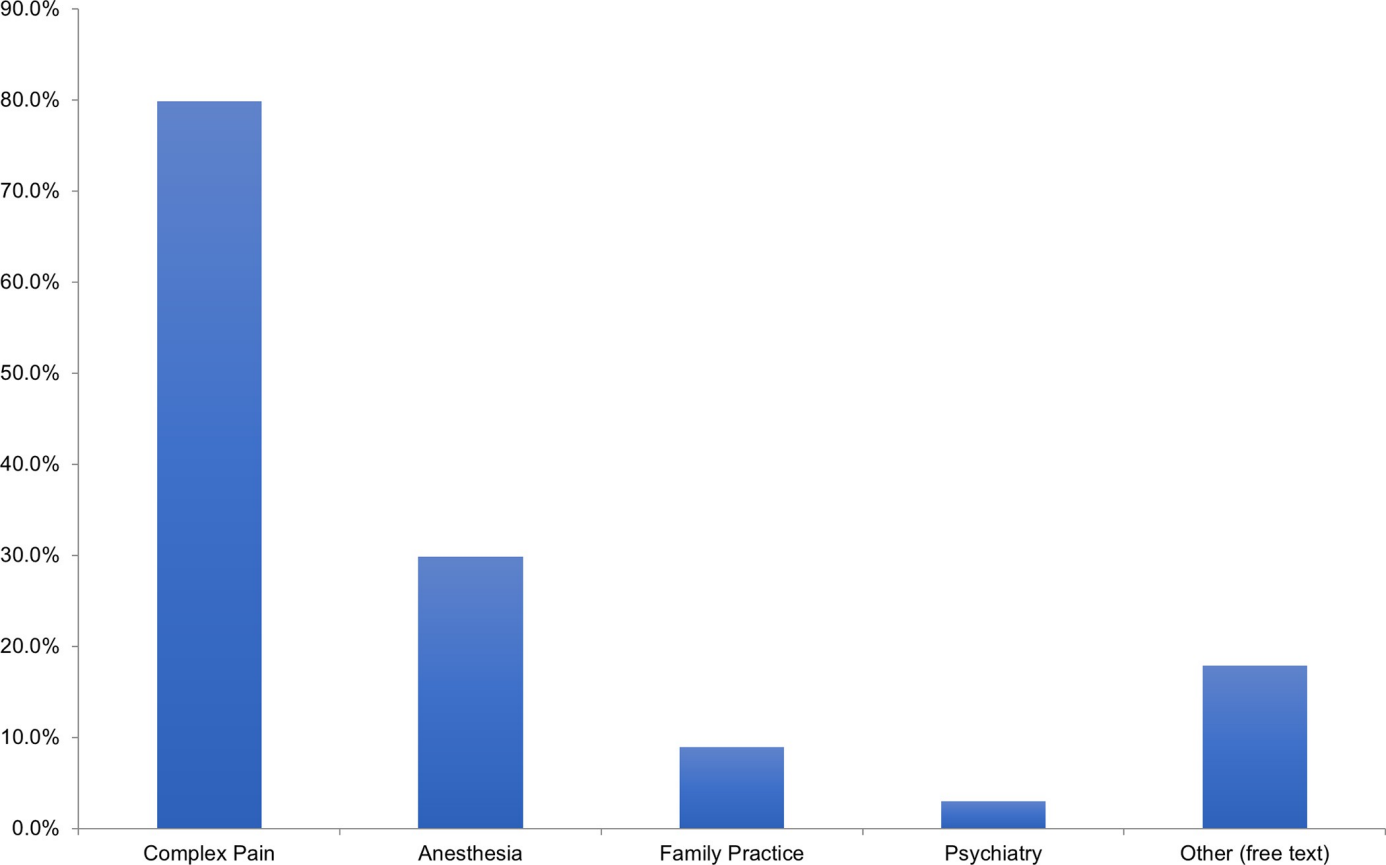
Other medical services typically involved for patients diagnosed with CRPS in distal radius fracture.

Only 19% of respondents offered routine prophylaxis for CRPS, but 40% of respondents did offer selective prophylaxis in patients whom they felt were high risk of developing CRPS. While most selected Vitamin C as their prophylactic agent of choice, pregabalin and gabapentin were both mentioned as additional prophylactic agents. In addition, early range of motion was commonly indicated as prophylaxis amongst respondents. When patients are diagnosed with CRPS after sustaining a distal radius fracture, the most common analgesic medications prescribed are acetaminophen and gabapentin (in 74% of respondents), followed by ibuprofen (Fig 3). The most common additional agents used for treatment include celecoxib (mentioned by 9 respondents (7%)) and corticosteroids (mentioned by 6 respondents (4%)). Occupational and physical therapy are the most frequently used additional treatment modalities in patients diagnosed with CRPS (Fig 4). 79% of respondents would involve their hospital’s Complex Pain service in this patient population (Fig 5).

**Fig 6:**
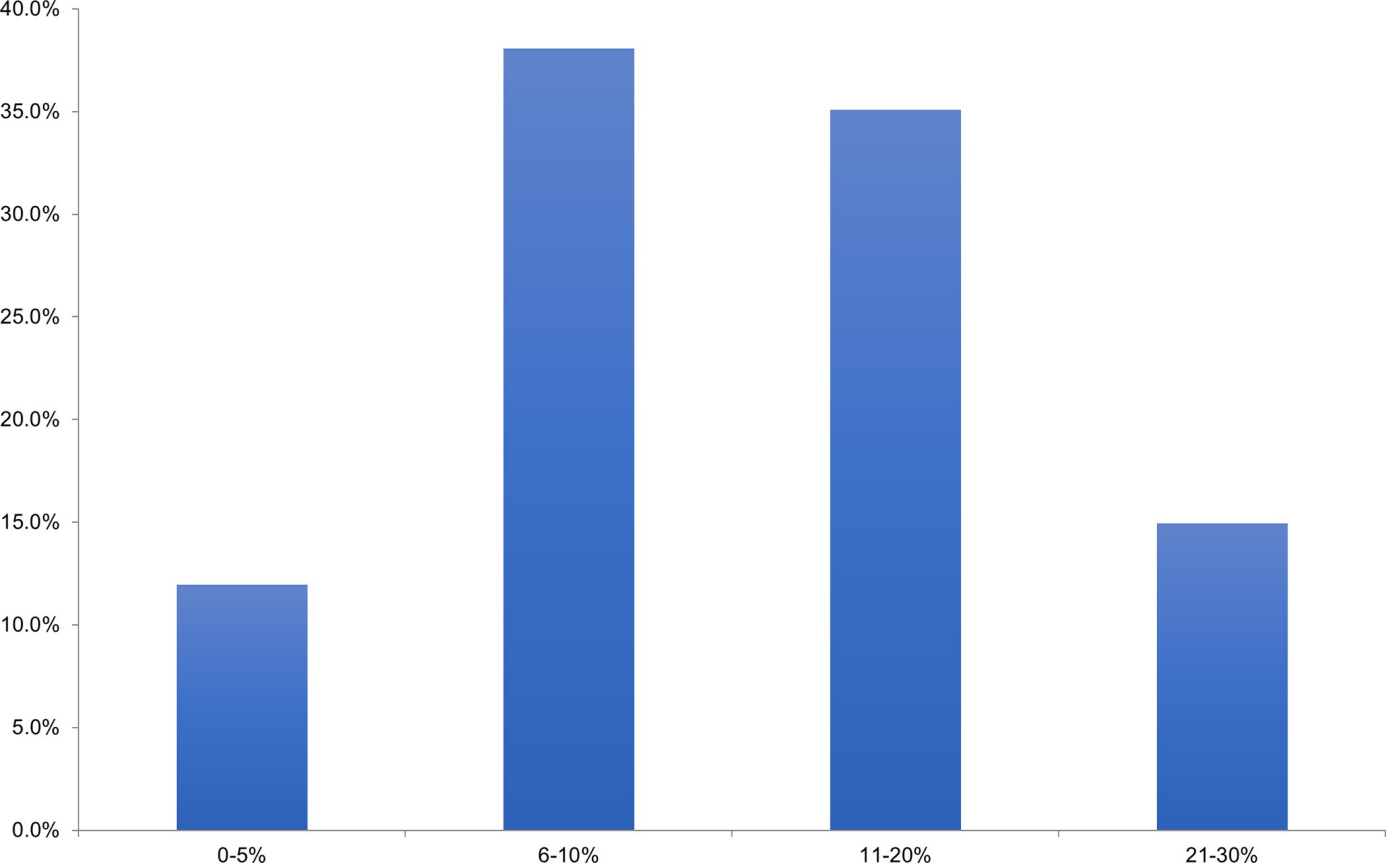
Absolute risk reduction of potential prophylactic agent for CRPS in distal radius fracture that would change surgeons’ practice.

**Fig 7:**
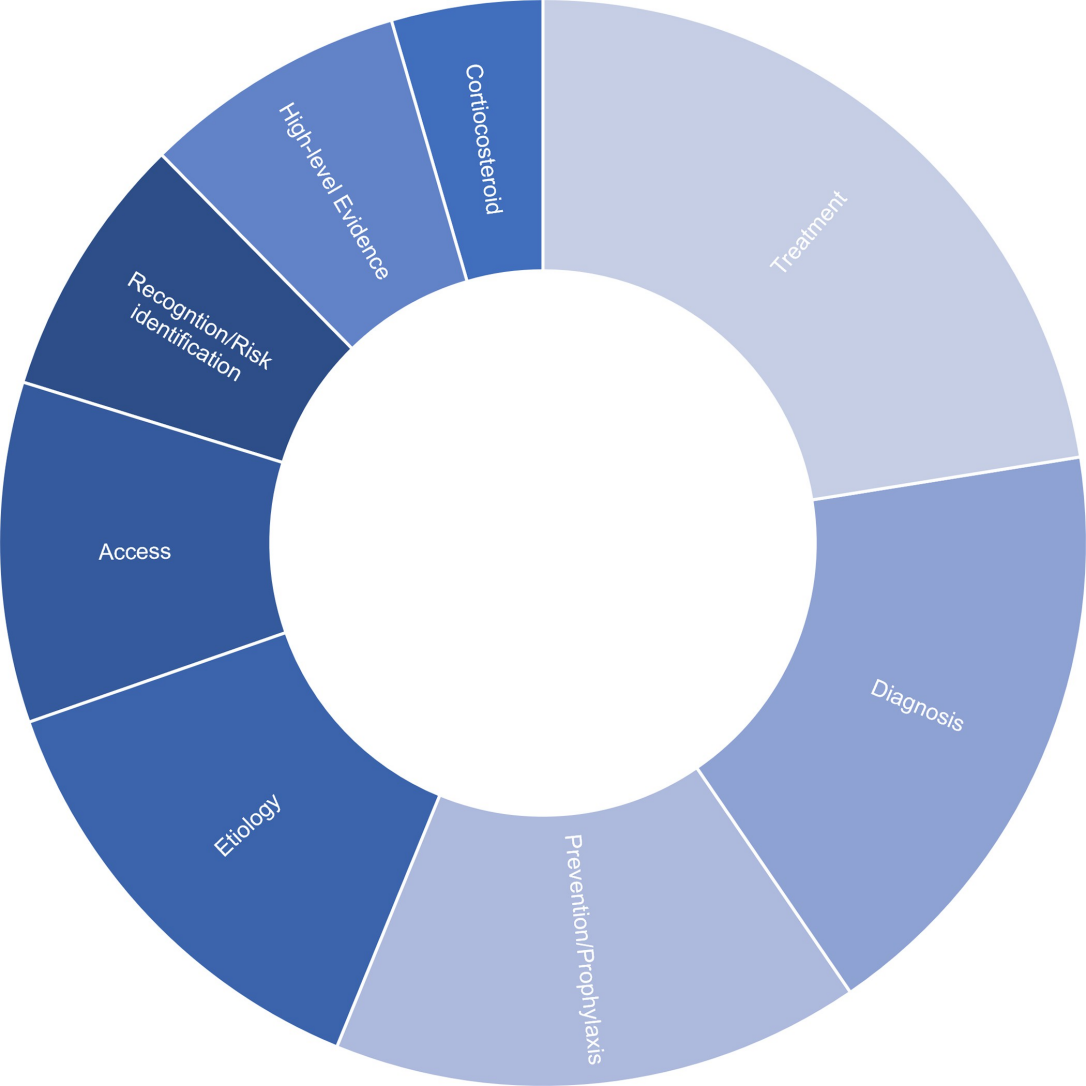
Common themes identified as clinical and research gaps in treating pain syndromes after distal radius fractures.

Amongst the surveyed surgeons, 66% felt neutral, uncomfortable, or very uncomfortable managing CRPS in distal radius fractures. When asked to consider adopting a prophylactic therapy, 38% of respondents indicated that a therapy that reduced the absolute risk of CRPS by 6–10% would be enough to change their practice (Fig 6). Common themes identified as gaps in current practice included a lack of evidence-based treatment and prevention strategies, diagnostic uncertainty, etiology, and available access to other treatment modalities and medical services (Fig 7).

## Discussion

In this survey, we captured the current practice preferences in distal radius fracture and CRPS management amongst a large population of surgeons who regularly treat these fractures in North America. From the self-reported demographics, an appropriate number of traumatologists and orthopaedic surgeons in varying subspecialties and practice settings have been included. While surgeons were overall consistent in their immobilization and activity restrictions for non-operatively treated distal radius fractures, we found that there were varying practices for mobilization in postoperative distal radius fractures. Range of motion and weightbearing of the affected extremity were started at different time points, ranging from 2 weeks post-op to between 6- and 12-weeks post-op. A study by Dennison et al. demonstrated that early range of motion and strengthening at 2 weeks after volar locked plating for distal radius fractures resulted in improved outcome scores earlier compared to those started at 5 weeks post-op [18]. Although ultimately there were no significant differences at 1 year, there was one case of CRPS in the late range of motion group out of 13 patients, compared to none in the 18 patients from the early group.

Throughout literature, the incidence of CRPS in distal radius fractures have a wide reported range [2–5]. This is likely due to the complexity and uncertainty of diagnostic criteria, leading to cases of over- and under-diagnosis. Although the Budapest Criteria for CRPS diagnosis was validated by Harden et al [17], it appears that in practice, the diagnosis is not as straightforward. In our study, we note that only 24% of the surgeons who responded utilized this set of criteria for CRPS diagnosis. Instead, various signs and symptoms associated with CRPS previously described (e.g., pain out of proportion, hypersensitivity, and skin changes) are still primarily used. It is possible that patients can be on a trajectory towards a diagnosis of CRPS, without exhibiting all the signs from the Budapest Criteria. It may be beneficial to act on early prevention or treatment of this condition, given the potential detrimental effects of disability, unemployment, and decrease in quality of life for the patient [6,7]. Having a reproducible and concise diagnostic criterion for CRPS that facilitates earlier diagnosis may enhance the management of this condition.

In the free text response section of this survey, many surgeons voiced that diagnostic uncertainty and difficulty with recognition and risk stratification remains a clinical challenge. Early high level of baseline pain (at least 5/10 on Visual Analogue Scale), dysynchiria (i.e., pain elicited to the affected limb when watching the unaffected limb in mirror), swelling, and slow reaction time have been shown to be predictors of CRPS [3]. In a study by Yeoh et al, depression was also found to be a predictor, with a Centre of Epidemiological Depression (CES-D) score of greater than or equal to 16 at baseline significantly correlating with higher rates of CRPS at 3 months post distal radius fracture [19]. However, the evidence in literature for these predictors is scarce, which was also identified in a recent systematic review by Rolls et al [20].

Despite the challenges of diagnosis and recognition of CRPS, it is interesting to note that 40% of surgeons who responded offered selective prophylaxis of CRPS in patients whom they identified as being high-risk. While Vitamin C was the most common prophylactic agent of choice, the literature around prophylaxis with Vitamin C for CRPS has been controversial. In a meta-analysis by Evaniew et al. on three randomized control trials that attempted to decipher this [12–14], no statistically significant effect was demonstrated for Vitamin C in the prevention of CRPS in distal radius fractures [16]. In terms of treatments for patients diagnosed with CRPS, our survey identified acetaminophen, gabapentin, and ibuprofen as the most common analgesic agents utilized. Although commonly used, in a Cochrane review by Wiffen et al, it was found that evidence for gabapentin in the management of CRPS was limited [21]. In addition, non-steroidal anti-inflammatory drugs, such as ibuprofen, have not been demonstrated to be effective in managing CRPS [22]. There have only been some anecdotal reports that showed cyclooxygenzse-2 inhibitors such as celecoxib to be possibly useful in CRPS, though there is caution to its use due to cardiac risks are not insignificant [23,24].

Several additional classes of pharmacological agents have been explored in the management of CRPS. Bisphosphonates have previously been shown to have some efficacy in early CRPS [25]; however, more recent trials did not confirm this [26]. Preliminary RCT data on carbamazepine showed analgesic efficacy in CRPS [27], though the side effect profile for this drug is not favourable and further research is lacking [23]. Tricyclic and heterocyclic antidepressants have been shown to be effective in neuropathic pain [28–30], but careful monitoring for side effects is required and there has not been clear evidence for use in CRPS [23]. There have been a few studies evaluating intravenous ketamine that demonstrated effective temporary relief in CRPS [31,32], though sample sizes were small and tolerance to ketamine and side effects are potentially problematic [23]. The role of opioids in management of CRPS is also indetermined, especially given the risk of dependence and overdose [23]. One RCT evaluating controlled-release morphine compared to placebo in CRPS demonstrated no significant difference in pain [27].

Alternatively, studies have identified glucocorticoids as potentially effective and safe for treating patients with CRPS, likely due to their anti-inflammatory and immune modulation properties [33–37]. One retrospective study of patients undergoing surgery for terrible triad injuries demonstrated improved elbow range of motion for patients receiving prophylactic intraoperative dexamethasone and 6-day oral tapering course of methylprednisolone compared to patients who did not, with no increased postoperative infection [38]. In our study, we found that some surgeons were using corticosteroids such as prednisone as additional agents to treat CRPS after distal radius fractures. In the free text response, we noted that surgeons purported anecdotal successes with use of steroids in managing CRPS in this population. As well, there were several comments with regards to interest in using steroids in this population and the need for further research. Therefore, further investigation to assess the role of glucocorticoids in CRPS management may be a relevant next step.

Besides pharmacological agents, the main treatment modalities and services sought for management of CRPS were occupational therapy, physiotherapy, and complex pain specialists. In a recent practice guideline on CRPS by Harden et al, occupational therapy, physical therapy, and a multi or interdisciplinary approach to management of CRPS were all recommended [23]. Occupational therapy offers guidance to patients through programs designed to minimize pain and edema, while maximizing functional use of the extremity [23]. Interventions such as mirror visual feedback therapy have been shown to be effective in early and intermediate CRPS, but not chronic CRPS [39,40]. On the other hand, physiotherapy has been shown to provide benefits to patients with CRPS using stress loading and isometric techniques [41,42], though high quality evidence is lacking [43]. Furthermore, psychological interventions such as exposure therapy in CRPS to target feared activities have shown improvements in pain intensity, pain catastrophizing, and disability at 6 months follow up [44,45]. In our study, several respondents voiced that the lack of access to multi-disciplinary services in their practice setting was a limiting factor in their management of this patient population. Therefore, it may be worthwhile to establish referral networks at sites where multi-disciplinary services are not readily available in order to support this patient population.

With the paucity of high-level evidence in management of CRPS in distal radius fractures, more vigorous research is required. Many surgeons are not fully comfortable with managing this complication and are interested in treatment and prophylactic agents that can reduce the rate of this devastating condition. This study does have some limitations. First, completion of the survey was voluntary and an element of response bias may exist. However, responses were anonymized and likely limits this. Second, our respondents represent a small sample of all practicing orthopaedic surgeons; other practice strategies and clinical concerns may not have been fully captured. As surveys were distributed to the COA and OTA, there were a higher percentage of trauma subspecialists, but there were almost a third of respondents who subspecialized in hand and wrist. It is possible that the estimation of the incidence of CRPS in distal radius fractures from the survey may not capture consistent experiences amongst the different orthopaedic subspecialists.

## Conclusions

Overall, this study highlights that the current practice amongst orthopaedic surgeons in the COA and OTA for CRPS in distal radius fractures is heterogeneous in diagnosis, treatment and prophylaxis strategies. A considerable portion of surgeons are not confident in their treatment of CRPS. Future studies using rigorous research methods are warranted to improve recognition and management of this challenging condition.

## Supporting information

S1 File Questions for survey of current CRPS practices. (PDF)

S1 Fig A) Primary practice setting and B) Primary orthopaedic practice amongst survey respondents.
(TIF)
