# Supplementary material for: Complex regional pain syndrome after distal radius fracture: A survey of current practices
Source: PLoS One. 2024 Nov 21;19(11):e0314307. doi: 10.1371/journal.pone.0314307 (PMC11581307; doi:10.1371/journal.pone.0314307)
Supplement: S1 File — (PDF) [file pone.0314307.s001.pdf]

Informed consent and agreement to participate in the survey

Project Title:
Complex regional pain syndrome after distal radius fractures – a survey of current practices

Research Team:
Alice Wang, MSc, MD, University of British Columbia; Email: alicew22@student.ubc.ca
David Stockton, MD, MASc, FRCSC, University of British Columbia; Email: David.Stockton@vch.ca (Principal Investigator)

Purpose:
The aim of this study is to survey surgeons in the COA and OTA on experiences and preferences in managing patients with distal radius fractures complicated by CRPS to better understand current practices.

Voluntary Participation: Participation is completely voluntary. If you wish to participate, please complete the survey. This should take approximately 10-15 minutes. You do not have to complete any questions that you do not wish to answer. Given the anonymous nature of the survey, once you have submitted your responses it will no longer be possible to withdraw them from the study. Granting agencies or journals may require survey data to be made available at the time of publication, with anonymity maintained.

Risks: There are no foreseeable risks involved in participating in this study other than those encountered in day-to-day life.

Benefits: Participants will receive no direct benefits from participating in this research survey. However, responses will help inform an in-depth understanding of current practice in management of CRPS in distal radius fractures and guide future research direction and practice.

Consent: By completing this survey, you are consenting to participate in this study.

Thank you for your consideration. Should you have any questions about the survey or research, please contact Dr. Alice Wang (alicew22@student.ubc.ca) or Dr. David Stockton (David.Stockton@vch.ca)

Ethics (H23-00644): This study has been reviewed by the UBC Research Ethics Board.

If you have any concerns or complaints about your rights as a research participant and/or your experiences while participating in this study, contact the Research Participant Complaint Line in the UBC Office of Research Ethics at 604-822-8598 or if long distance e-mail RSIL@ors.ubc.ca or call toll free 1-877-822-8598.

Survey Questions

1. What is your practice setting? (Select all that apply)
   a. Private
   b. Tertiary hospital with academic affiliation
   c. Community/rural hospital

2. What is your primary orthopaedic practice? (Select all that apply)
   a. General
   b. Trauma
   c. Arthroplasty
   d. Hand/wrist
   e. Elbow/shoulder
   f. Foot and ankle
   g. Knee and sports
   h. Spine
   i. Pediatric

3. When treating distal radius fractures non-operatively, what is your mode of immobilization?
   a. Circumferential cast
   b. Splint
   c. Other (free text)

4. When treating distal radius fractures operatively, what is your mode of immobilization immediately post-op?
   a. Circumferential cast
   b. Splint
   c. Simple dressings

5. When treating distal radius fractures <u>non-operatively</u>, when do you typically allow patients to start partial weight-bearing (eg. Perform daily ADLs) with that extremity?
   a. At 2 weeks post injury
   b. Between 2 and 6 weeks post injury
   c. At 6 weeks post injury
   d. Between 6 and 12 weeks post injury

6. When treating distal radius fractures <u>non-operatively</u>, when do you typically allow patients to start full weight-bearing (no activity restrictions) with that extremity?
   a. At 2 weeks post injury
   b. Between 2 and 6 weeks post injury
   c. At 6 weeks post injury
   d. Between 6 and 12 weeks post injury

7. When treating distal radius fractures operatively, when do you typically allow patients to start partial weight-bearing (eg. Perform daily ADLs) with that extremity?
    a. At 2 weeks post injury
    b. Between 2 and 6 weeks post injury
    c. At 6 weeks post injury
    d. Between 6 and 12 weeks post injury

8. When treating distal radius fractures operatively, when do you typically allow patients to start full weight-bearing (no activity restrictions) with that extremity?
    a. At 2 weeks post injury
    b. Between 2 and 6 weeks post injury
    c. At 6 weeks post injury
    d. Between 6 and 12 weeks post injury

9. When treating distal radius fractures non-operatively, when do you typically allow patients to start range of motion exercises?
    a. At 2 weeks post injury
    b. Between 2 and 6 weeks post injury
    c. At 6 weeks post injury
    d. Between 6 and 12 weeks post injury

10. When treating distal radius fractures operatively, when do you typically allow patients to start range of motion exercises?
    a. At 2 weeks post-op
    b. Between 2 and 6 weeks post-op
    c. At 6 weeks post-op
    d. Between 6 and 12 weeks post-op

11. From your practice experience, what is the estimated incidence of CRPS in distal radius fractures?
    a. <1%
    b. 1-10%
    c. 11-20%
    d. 21-30%
    e. 31-40%
    f. 41-50%
    g. >50%

12. What features do you use to diagnose CRPS in distal radius fractures? (Select all that apply)
    a. Budapest Criteria
    b. International Association for the Study of Pain Criteria

c. Pain out of proportion to the inciting event
d. Hypersensitivity to light touch or temperature
e. Temperature or colour asymmetry between limbs
f. Edema/sweating asymmetry between limbs
g. Trophic skin changes (hair, nail, skin)
h. Motor dysfunction (weakness, decreased range of motion)

13. Do you offer routine prophylaxis for CRPS in distal radius fractures for all of your patients?
    a. Yes
    b. No

14. If you answered "yes", what routine prophylactic agent do you use?
    a. N/A
    b. Vitamin C
    c. Other (free text)

15. Do you offer selective prophylaxis for CRPS in distal radius fractures patients whom you feel are high risk?
    a. Yes
    b. No

16. If you answered "yes", what selective prophylactic agent do you use?
    a. N/A
    b. Vitamin C
    c. Other (free text)

17. What typical post-op pain regimen do you prescribe for operatively treated distal radius fractures? (Select all that apply)
    a. Acetaminophen
    b. Ibuprofen
    c. Ketorolac
    d. Opioids
    e. Gabapentin/pregabalin
    f. Other (free text)

18. What analgesic medications do you prescribe patients diagnosed with CRPS in distal radius fractures? (Select all that apply)
    a. Acetaminophen
    b. Ibuprofen
    c. Ketorolac
    d. Opioids
    e. Gabapentin/pregabalin
    f. Other (free text)

19. What additional treatment modalities do you offer or refer for patients diagnosed with CRPS in distal radius fractures? (Select all that apply)
    a. None
    b. Occupational/physical therapy
    c. Parenteral agents (eg. Lidocaine, corticosteroids)
    d. Psychological treatments (eg. Counselling, adaptive therapy)
    e. Corticosteroids
    f. Anti-depressants
    g. Anticonvulsants
    h. Calcium channel blockers
    i. Other (free text)

20. What other medical services do you typically involve for patients diagnosed with CRPS in distal radius fractures? (Select all that apply)
    a. Anesthesia
    b. Complex Pain
    c. Family Practice
    d. Psychiatry
    e. Other (free text)

21. What is your comfort level in managing CRPS in distal radius fractures?
    a. 5 – Very comfortable
    b. 4 – Comfortable
    c. 3 – Neutral
    d. 2 – Uncomfortable
    e. 1 – Very uncomfortable

22. If a therapy or pharmacologic agent was investigated for prophylaxis of CRPS in distal radius fractures, what would the absolute risk reduction (ARR) have to be in order for you to change your practice? For example, if the incidence was 30%, would you change your practice if a therapy decreased the incidence to 20% (ARR = 10%)?
    a. 0-5%
    b. 6-10%
    c. 11-20%
    d. 21-30%

23. From your experience, what clinical gaps exist when treating pain syndromes after distal radius fractures?
    (free text)
