# Supplementary figures and images for: Complex regional pain syndrome after distal radius fracture: A survey of current practices
Source: PLoS One. 2024 Nov 21;19(11):e0314307. doi: 10.1371/journal.pone.0314307 (PMC11581307; doi:10.1371/journal.pone.0314307)

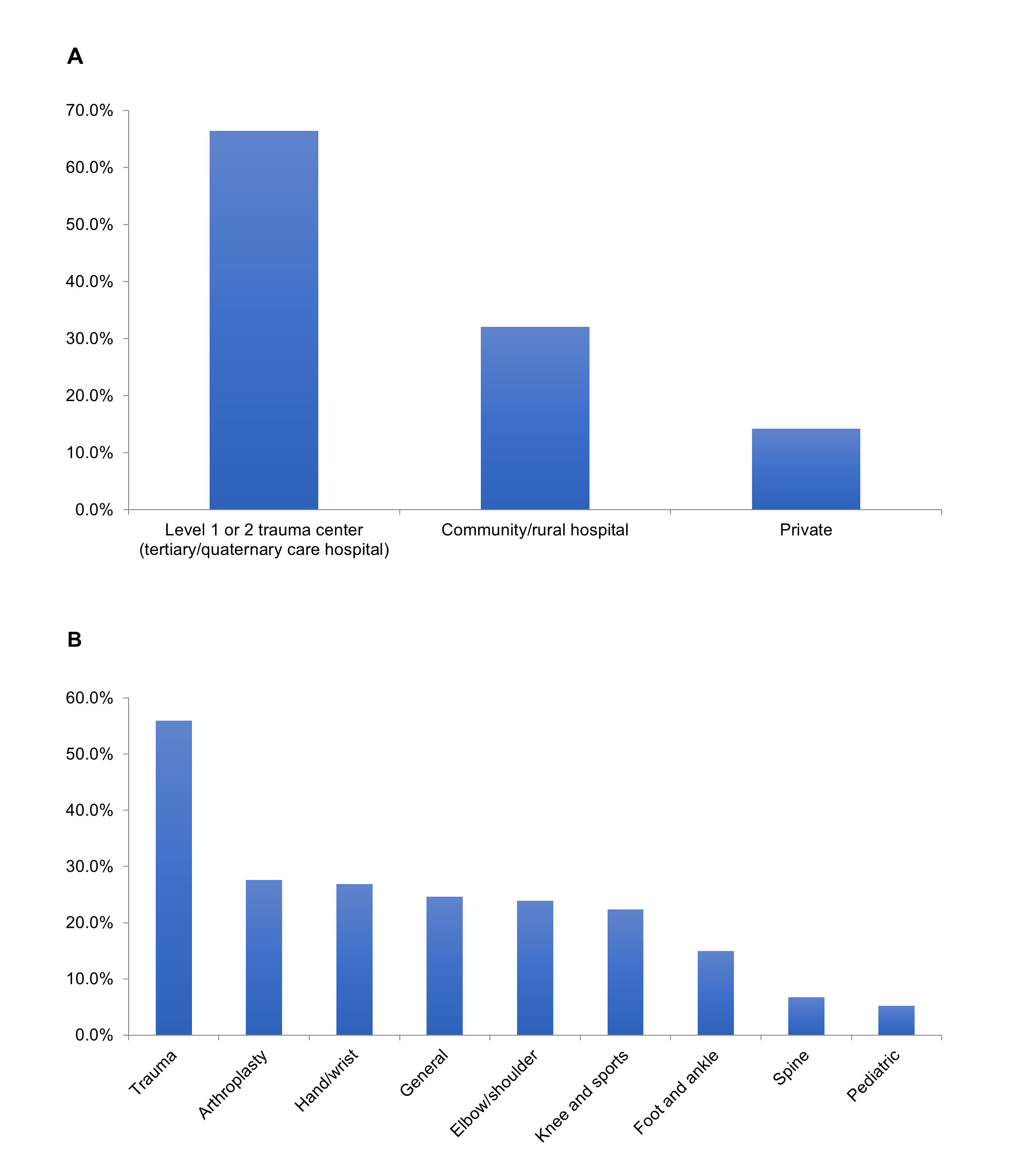

Supplement: S1 Fig — A) Primary practice setting and B) Primary orthopaedic practice amongst survey respondents. (TIF) [file pone.0314307.s002.tif]
